# Salamanders and other amphibians are aglow with biofluorescence

**DOI:** 10.1038/s41598-020-59528-9

**Published:** 2020-02-27

**Authors:** Jennifer Y. Lamb, Matthew P. Davis

**Affiliations:** 0000 0001 0738 3196 grid.264047.3 St. Cloud State University, Department of Biology, St. Cloud, Minnesota, 56301 USA

**Keywords:** Biodiversity, Evolution, Herpetology

## Abstract

Biofluorescence is the absorption of electromagnetic radiation (light) at one wavelength followed by its reemission at a lower energy and longer wavelength by a living organism. Previous studies have documented the widespread presence of biofluorescence in some animals, including cnidarians, arthropods, and cartilaginous and ray-finned fishes. Many studies on biofluorescence have focused on marine animals (cnidarians, cartilaginous and ray-finned fishes) but we know comparatively little about the presence of biofluorescence in tetrapods. We show for the first time that biofluorescence is widespread across Amphibia, with a focus on salamanders (Caudata), which are a diverse group with a primarily Holarctic distribution. We find that biofluorescence is not restricted to any particular family of salamanders, there is striking variation in their fluorescent patterning, and the primary wavelengths emitted in response to blue excitation light are within the spectrum of green light. Widespread biofluorescence across the amphibian radiation is a previously undocumented phenomenon that could have significant ramifications for the ecology and evolution of these diverse and declining vertebrates. Our results provide a roadmap for future studies on the characterization of molecular mechanisms of biofluorescence in amphibians, as well as directions for investigations into the potential impact of biofluorescence on the visual ecology and behavior of biofluorescent amphibians.

## Introduction

Biofluorescence occurs when higher energy wavelengths of light (e.g., ultra-violet or blue light) are absorbed and subsequently reemitted at lower energy wavelengths in living organisms, resulting in a glow with brilliant fluorescent colors including blues, greens, and reds. This phenomenon is widespread among animals, most notably within the cnidarians, arthropods, and cartilaginous and ray-finned fishes^1–5^, but it has also been documented in lineages of terrestrial animals^6^. Biofluorescence may be initiated by abiotic (e.g., sunlight) or biotic sources of light. Many surveys have documented biofluorescence in response to excitation by ultra-violet^6–13^ and visible blue light^2–4,14–16^. Mechanisms which produce biofluorescent light vary and can involve proteins (e.g., green fluorescent protein), pigments, metabolites, or mineralization^1,2,5,8,10,12,13,16^, but in most lineages of animals the exact mechanisms are unknown. Hypothesized functions for biofluorescence include communication^5,13^, sexual selection^7^, camouflage^3,13^, and improved visual acuity^9,11,14^ to perhaps no function at all in some lineages^5^.

We know comparatively less about the occurrence of biofluorescence in tetrapods than we do for other vertebrates such as cartilaginous and ray-finned fishes^3,16^, and most surveys which include tetrapods focus on fluorescence in response to ultra-violet radiation. Biofluorescence under ultra-violet light has been documented in chameleons^10^, parrots^7^, penguins^8^, some rodents^6,13^, as well as in a handful of amphibians^9,11,12^. Ultra-violet light attenuates rapidly with depth in both freshwater and marine environments whereas blue light can penetrate further^17^. Among tetrapods, biofluorescence in response to ambient blue light has only been documented in marine turtles^15^. The ambient light environment is more complex in terrestrial and freshwater ecosystems than in marine systems, but in some cases blue light can dominate^17,18^. Whether other tetrapods fluoresce in response to light in the visible blue spectrum is unknown.

In this study, we present the first taxonomically comprehensive exploration of biofluorescence in amphibians, with a focus on the salamanders and newts (Caudata). Salamanders are the second most diverse lineage of amphibians, with 737 species^19^, many of which are threatened or endangered^20,21^. Salamanders occur across a myriad of freshwater and terrestrial ecosystems and utilize both chemosensory and visual cues in their environment^22^. Prior to this work biofluorescence had only previously been documented in one species of salamander^11^ in response to ultra-violet light. Whether biofluorescence is present and may play a role in the ecology and evolution of other salamander lineages is unknown.

## Results

**Figure 1:**
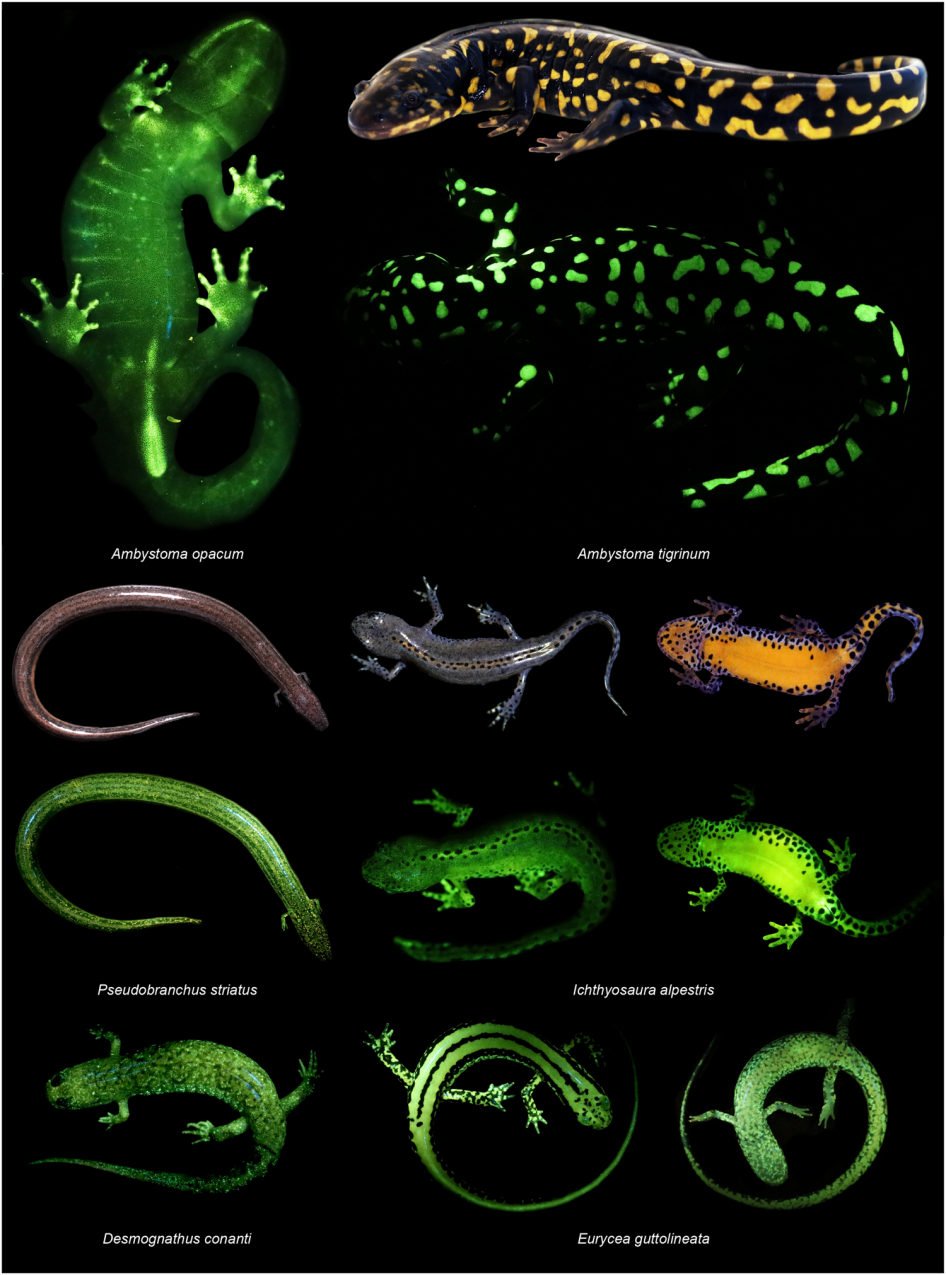
Biofluorescent patterns are variable across salamander diversity and anatomy. We observed biofluorescence across the salamander radiation. Salamanders with bold patterns and colors fluoresce brightly (e.g., *Ambystoma tigrinum*), and dorsal surfaces often fluoresce less intensely than ventral surfaces (e.g., *Icthyosaura alpestris*) depending on patterning. In some salamanders (e.g., *Ambystoma* *opacum*) bones (e.g., dentary, digits) that are otherwise not visible under white light fluoresce distinctly, as does the cloacal region. Included are white light images of three species (*A*. *tigrinum*, *Pseudobranchus striatus*, *I*. *alpestris*) above images depicting biofluorescence. Biofluorescence was imaged by exposing individuals to blue light (440–460 nm) and viewing them through a yellow long pass filter (500 nm).

**Figure 2:**
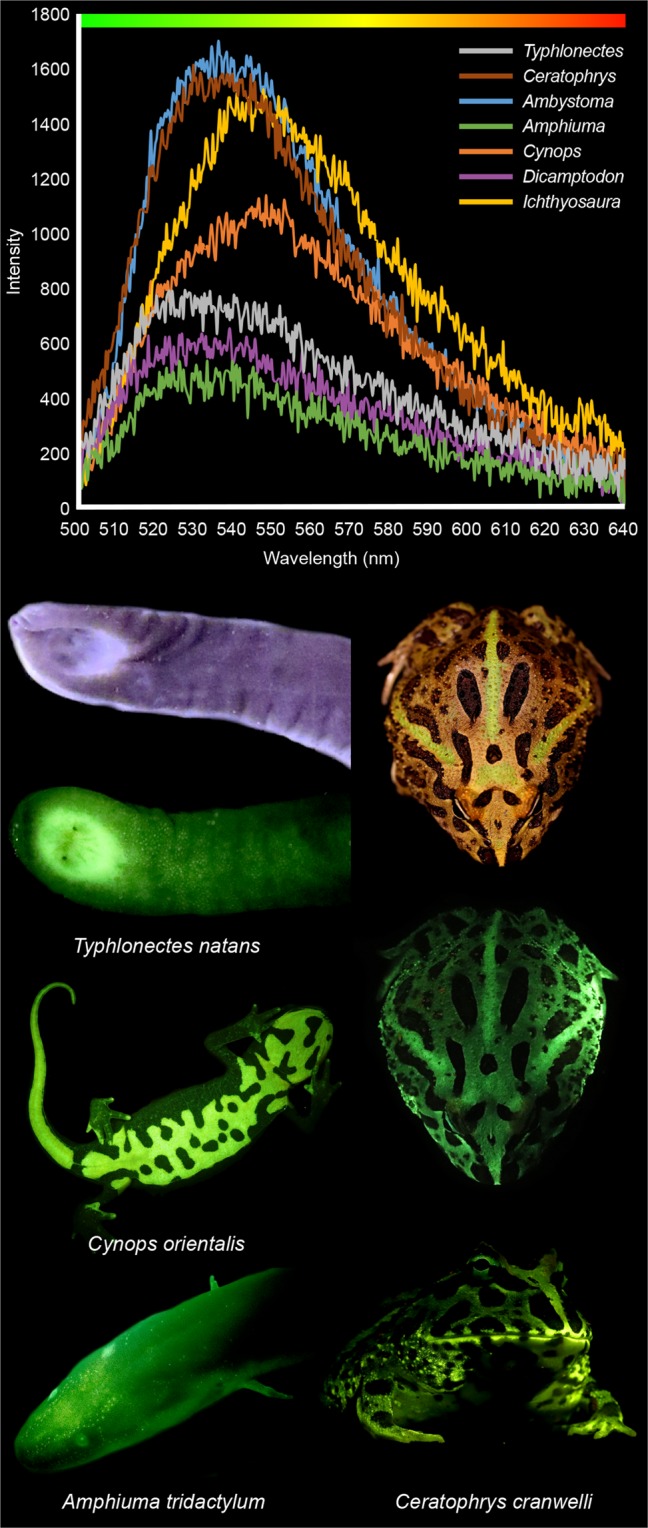
Amphibian biofluorescent emission spectra. The peak emission wavelengths of the biofluorescent light emitted by salamanders, frogs (*Ceratophrys cranwelli*), and caecelians (*Typhlonectes natans*) are green to greenish-yellow. Biofluorescent emissions were measured with a FLAME spectrometer through a yellow long pass filter (500 nm) from either the dorsal (*Ceratophrys*, *Ambystoma*, *Dicamptodon*) or ventral surfaces (*Typhlonectes*, *Amphiuma*, *Cynops*, *Icthyosaura*), but we did not focus on any specific part of the anatomy (see Methods for further detail). Relative intensities of these biofluorescent emissions varied substantially across taxa. Pictured are white light images of *T*. *natans* and *C*. *cranwelli* above images of these species biofluorescing. Biofluorescence was imaged by exposing individuals to blue light (440–460 nm) and viewing them through a yellow long pass filter (500 nm).

Amphibians fluoresce green to yellow in response to blue (440–460 nm) (Figs. 1 and 2) and ultra-violet excitation light (360–380 nm) (Supplementary Fig. 2), but the biofluorescent light emitted under blue excitation is more intense than when excited by ultra-violet light (Supplementary Fig. 2). Fluorescent green coloration in response to blue excitation light is strikingly widespread across the amphibian radiation (Figs. 1 and 2) and is the focus of this survey. Every amphibian species and life stage we examined, including aquatic larvae, is biofluorescent (Supplementary Table 1). Peak fluorescent emissions coming from these amphibians (Fig. 2) fall within the spectrum of green light (ca. 520–560 nm). The intensities of fluorescent light we recorded were variable among taxa (Figs. 1 and 2) and weakest for those that lacked bright or reflective pigments (i.e., yellows, oranges, whites). Sample sizes for each species included one to five individuals (Supplementary Table 1), however additional sampling is needed to make intraspecific comparisons between individuals, sexes, or life stages. Our survey included representatives from eight of ten families of salamanders, five families of frogs, and one family of caecilians (Supplementary Table 1).

Biofluorescent patterns differ substantially among amphibians (Figs. 1 and 2). Areas with concentrated pigments, such as the yellow blotches of eastern tiger salamanders (*Ambystoma tigrinum*, Fig. 1), yellow stripes of three-lined salamanders (*Eurycea guttolineata*, Fig. 1), and orange venters of alpine (*Icthyosaura alpestris*, Fig. 1) and Chinese fire-belly newts (*Cynops orientalis*, Fig. 2), are a striking fluorescent green or green-orange. Scattered chromatophores and white iridophores fluoresce green in taxa like the northern dwarf siren (*Pseudobranchus striatus*, Fig. 1), spotted dusky salamander (*Desmognathus conanti*, Fig. 1), and the three-lined salamander (*E*. *guttolineata*, Fig. 1). Salamander species that lack prominent markings, such as the three-toed amphiuma (*Amphiuma tridactylum*, Fig. 2), also fluoresce. In some salamanders, the ventral surface fluoresces more intensely than the dorsal surface (*I*. *alpestris*, Fig. 1; *C*. *orientalis* and *A*. *tridactylum*, Fig. 2), and in other amphibians specific parts of the anatomy, or secretions (Supplementary Fig. 3), also fluoresced. The iridescent peritonea of larval ambystomatid salamanders, bones in the digits of the marbled salamander (*Ambystoma opacum*, Fig. 1), and the cloacal regions of both the marbled salamander (*A*. *opacum*, Fig. 1) and the caecilian (*Typhlonectes natans*, Fig. 2) exhibited prominent fluorescence. We observed that mucous-like secretions from the skin fluoresced green in some groups (i.e., Ambystomatidae, Amphiumidae, Typhlonectidae), as did urine (i.e., Dicamptodontidae) (Supplementary Fig. 3).

**Figure 3:**
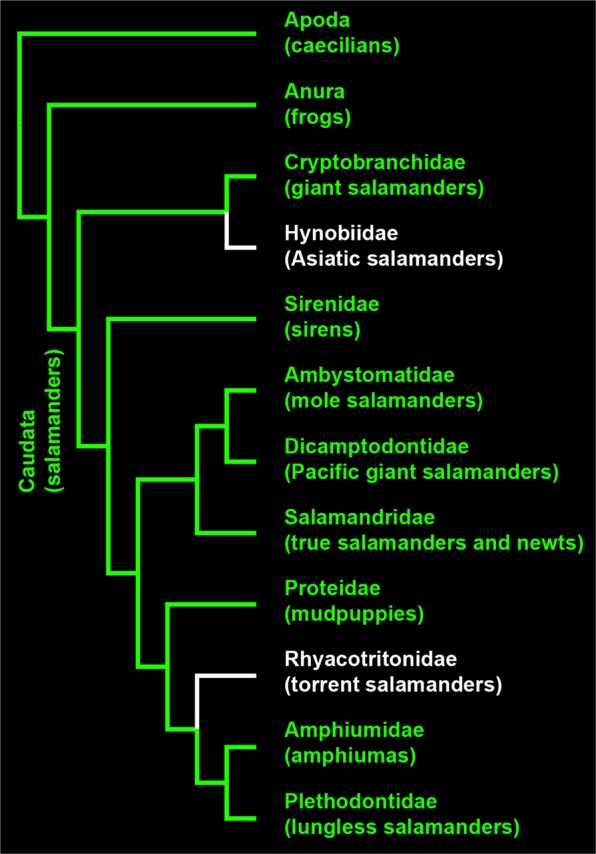
Distribution of fluorescence across amphibians. The evolutionary relationships among Amphibians with an emphasis on salamander (Caudata) families are presented based on the hypothesis of relationships from Pyron and Wiens^23^ inferred from gene-fragment data. Green branches and terminals indicate lineages where taxonomic representatives have been confirmed to have biofluorescence in this study. White terminals and branches indicate that biofluorescence is currently unknown in those taxa.

When examined in the context of a taxonomically robust hypothesis of evolutionary relationships for amphibians^23^, we can infer that fluorescence in response to blue excitation light is not restricted to a particular lineage of salamanders and that it is likely present throughout Caudata (Fig. 3, Supplementary Fig. 1). The presence of fluorescence in caecilians and in disparate lineages of frogs (Fig. 3, Supplementary Table 1) suggests that biofluorescence is taxonomically widespread in those radiations, and that biofluorescence likely appeared early in the evolutionary history of amphibians.

## Discussion

There could be multiple proximate causes for fluorescence in response to blue or ultra-violet wavelengths within amphibians and the exact mechanisms that produce biofluorescence in these vertebrates require further study. Biofluorescence associated with bold colors could be the result of both chemical and structural elements of the amphibian dermal chromatophore unit^24^. Some pigments, like pterins and carotenoids, or reflective structures containing guanine have been shown to fluoresce^5,9,25^. Both are present in chromatophores or elsewhere in the skin of larval and metamorphosed amphibians^24,25^.

Alternatively, there could be sources of fluorescence in salamanders and other amphibians independent of their pigmentary systems. Green fluorescent proteins and their analogs are responsible for fluorescence in some invertebrates (e.g., cnidarians)^1^ and vertebrates (e.g., eels)^2^, but similar proteins have not yet been characterized from amphibians. Fluorescent compounds called hyloins have been documented from Neotropical tree frogs (Hylidae) and are associated with their lymph and glandular secretions^9^. Similar compounds may be responsible for the biofluorescent, mucous-like secretions which we observed in salamanders and caecilians (Supplementary Fig. 3). Recently, fluorescence in swell sharks^4^ in response to blue light has been attributed to a newly discovered fluorescent metabolite in their skin^16^. For other vertebrates, ossified elements immediately beneath the skin are responsible for biofluorescent patterns (e.g., chameleons^10^ and pumpkin toadlets^12^) under ultra-violet excitation. Here we found that the bones in the digits of the marbled salamander (*A*. *opacum*, Fig. 1) fluoresced in response to blue light.

Whether the biofluorescent light produced by an amphibian is perceived by conspecifics or heterospecifics depends in part on if individuals are active under the conditions necessary for fluorescence^5^. Amphibians occupy a variety of habitats, often moving between terrestrial and freshwater systems, and the ambient light environments they experience are complex^17^. In forests ambient light varies with the structure of the vegetative community, weather, and time of day^17,18,26^, but several forest types contain patches of habitat in which blue wavelengths are prevalent^18^. Many amphibians, including salamanders, are crepuscular or nocturnal. During twilight, the ambient spectra in terrestrial systems shifts to predominantly blue light^18,26^. Both light environments include wavelengths in the visible blue spectrum which we have shown result in green fluorescence (Fig. 2). Terrestrial organisms that are active on the surface during daylight and early twilight will also be exposed to ultra-violet radiation^17^ which can result in biofluorescence in both anurans^9,12^ and salamanders^11^. We have added three species to the list of amphibians which biofluoresce in response to ultra-violet excitation (*Ceratophrys cranwelli*, *Ambystoma tigrinum*, *A*. *laterale*) (Supplementary Fig. 2), though the intensity of fluorescence we observed was less than when exposed to blue excitation light (Figs. 1 and 2). Although our study and others have confirmed the presence of fluorescence in anurans in response to ultra-violet light, other recent work surveying for biofluorescence in Neotropical tree frogs did not identify strong emissions under ultra-violet excitation^27^.

Perception of biofluorescent light also depends on the sensitivity of the eye of a potential observer. Rod photoreceptors in the retina play a key role in vision during low-light conditions^26^ such as those during twilight. Like other vertebrates, salamanders, frogs, and caecelians have “red-rods” which are maximally sensitive to green light^22,28–30^. Biofluorescence in amphibians could potentially contribute to achromatic vision and perception of other individuals in low-light environments. Recent studies demonstrate that the unique, dual-rod system of some amphibians, which includes a “green rod” that is maximally sensitive to blue light^22,31^, allows for color discrimination in dim-light^31^ and biofluorescence may contribute to color perception in those environments for some species. “Green rods” are absent in some salamanders (e.g., some salamandrids^30^) but present in others (e.g., ambystomatids^29^).

Whether biofluorescence contributes dramatically to the overall light emitted from an organism may vary across taxa. Taboada *et al*.^9^ documented that biofluorescence in response to ultra-violet light contributes between 18 and 29% of the total light emitted from some species of Neotropical tree frogs (Family Hylidae) under natural, dimly lit conditions. In contrast, Goutte *et al*.^12^ found that biofluorescent emissions derived from bony elements in pumpkin toadlets (Family Brachycephalidae) contributed to less than 3% of the total light emitted by these anurans, and that in this case fluorescent signals were likely negated by ultra-violet light being reflected by the toadlets. In our survey, both the blue and ultra-violet light sources used were more intense than would be experienced by these organisms *in situ*, and the contributions from biofluorescence versus reflectance under natural conditions is in need of further study across the range of habitats amphibians occupy.

In tetrapods, as in other vertebrates, biofluorescence may function in both intra- and interspecific communication and crypsis. In flying squirrels fluorescence is hypothesized to aid in camouflage against a backdrop of lichens emitting similar fluorescent spectra, or potentially in Batesian mimicry of co-occurring predatory owls with similar biofluorescent profiles^13^. Biofluorescent plumage in parrots functions in sexual selection and mate choice^7^, whereas in anurans it may enhance the brightness of individuals making them easier to detect by conspecifics^9^. Biofluorescence in salamanders may serve similar functions, and salamanders with complex reproductive behaviors involving visual signals (e.g., newts [Salamandridae] and lungless salamanders [Plethodontidae]) are observed to biofluoresce in this study. Cloacal biofluorescence in some species is particularly intriguing (Fig. 1) as this region is often the target of investigative behaviors during courtship in salamanders. The peak emissions produced by amphibians observed in this study fall within the range of green light (Fig. 2), a color which would set them apart from background vegetation that fluoresces yellow or red under blue excitation light. Future ethological, anatomical, and chemical studies are needed to determine the functional roles, if any, biofluorescence serves in the biology and evolution of amphibians.

Biofluorescence is widespread and variable across Amphibia, and our findings shine a new light on how much more we have to learn about the biology of these fascinating vertebrates. Our study provides a roadmap for future efforts intent on exploring biofluorescence in amphibians, from the potential ramifications of fluorescence on their ecology, to the chemical or structural mechanisms contributing to this phenomenon, to the potential broad applications of this knowledge across scientific disciplines (e.g., developmental biology, medical fields). Biofluorescence may also prove a useful tool in documenting amphibian biodiversity in complex microenvironments. Small, cryptically colored, and/or nocturnally active species can be hard to locate among leaf litter or dense vegetation. We propose that scientists documenting the biodiversity of amphibians could use excitation light devices and filtering lenses to visualize fluorescent amphibians and improve their ability to find taxa which are otherwise difficult to detect. This could be an inexpensive and transformative approach to how we survey the biodiversity of amphibians worldwide.

## Methods

We had approval to obtain and/or observe specimens from the field (Permits: Minnesota Department of Natural Resources 28984, 201939, 2019-17R), pet trade, and aquaria (Shedd Aquarium, Chicago, IL). All work with animals was conducted with accordance to appropriate St. Cloud State University Institute for Animal Care and Use Committee protocols (SCSU IACUC 17–112) for handling, imaging, and examining amphibians for fluorescence. We imaged from one to five live or freshly dead specimens with a DSLR camera (Cannon EOS Rebel T7i, Canon Mark IV) in combination with a 60- or 100-mm macro lens, or with a digital camera (Olympus TG-5). Specimen digitization included the use of a black background with white light illumination. We followed the protocol of Sparks *et al*.^3^ to survey for biofluorescence and illuminated specimens with a blue excitation light (440–460 nm; NIGHTSEA DFP-1 Flashlight or gooseneck lamps) in combination with a NIGHTSEA longpass filter (500 nm). Critically endangered taxa (i.e., *Cryptobranchus allenganiensis*) were examined alive within their exhibits at the Shedd Aquarium (Chicago, IL) with the aid of aquarium personnel. We additionally surveyed for fluorescence in response to an ultra-violet excitation light (360–380 nm; NIGHTSEA gooseneck lamps) in combination with a NIGHTSEA longpass filter (415 nm) in a limited number of taxa. These included one individual each of one species of frog (*Ceratophrys cranwelli*) and two species of salamanders (*Ambystoma tigrinum*, *A*. *laterale*).

We used a FLAME-S-VIS-NIR-ES spectrometer (OceanOptics) with a 600 um UV/VIS fiber optic probe and a UV/VIS 200–2000 nm collimating lens in combination with a longpass NIGHTSEA filter (500 nm) to measure the spectra of biofluorescent emissions produced by a subset of live amphibians during exposure to blue-light (440–460 nm). Amphibians were held in an acrylic photobox or glass containers with black backgrounds in a dark environment while we collected spectra recordings in the proprietary OceanView program. We collected 10 independent spectra recordings per individual from the dorsal and/or ventral surfaces and we attempted to standardize our efforts by collecting recordings with maximum intensities that were less than 2000 pixel counts. These independent readings were then then averaged to generate the spectra profiles for representative amphibians for each surface (i.e., dorsal and ventral)(Fig. 2).

We mapped the presence or absence of biofluorescence across the taxa sampled onto a taxonomically comprehensive phylogeny of salamander relationships (Pyron and Wiens^23^) inferred from gene-fragment data (Fig. 3, Supplementary Fig. 1). The hypothesis of evolutionary relationships from Pyron and Wiens^23^ is presented herein trimmed to a single representative for Apoda (caecilians) and Anurans (frogs), and a representative for each family (Fig. 3) and genus (Supplementary Fig. 1) within Caudata (salamanders).

## Supplementary information

Supplementary materials.
