# Supplementary material for: Salamanders and other amphibians are aglow with biofluorescence
Source: Sci Rep. 2020 Feb 27;10:2821. doi: 10.1038/s41598-020-59528-9 (PMC7046780; doi:10.1038/s41598-020-59528-9)

Supplementary figures and tables for:

Salamanders and other amphibians are aglow with biofluorescence

Jennifer Y. Lamb[1*] and Matthew P. Davis[1*].

[1]St. Cloud State University, Department of Biology, St. Cloud, Minnesota 56301, USA.

* corresponding authors: jylamb@stcloudstate.edu; mpdavis@stcloudstate.edu

Supplementary Table 1. Species of amphibians surveyed for biofluorescence in response to blue excitation wavelengths. We observed biofluorescence across all 32 species of amphibians we tested. Our survey included eight families of salamanders, five families of frogs, and one family of caecilian. We were not able to survey the larval forms for each of these species but did confirm that the larvae of six species also fluoresced. The number of individuals indicated is the number of individuals tested for fluorescence when exposed to blue excitation light (440 – 460 nm).

Amphibian Clade	Family	Genus	Species	Common Name	Fluorescence	Life-history Stage	Number of Individuals
Anura	Bufonidae	*Anaxyrus*	*americanus*	American toad	Present	Metamorphosed	2
Anura	Bufonidae	*Anaxyrus*	*fowleri*	Fowler's toad	Present	Metamorphosed	1
Anura	Bufonidae	*Anaxyrus*	*terrestris*	Southern toad	Present	Metamorphosed	1
Anura	Ceratophrydae	*Ceratophrys*	*cranwelli*	Green pac-man frog	Present	Metamorphosed	1
Anura	Dendrobatidae	*Epipedobates*	*anthonyi*	Phantasmal dart frog	Present	Metamorphosed	1
Anura	Hylidae	*Cruziohyla*	*craspedopus*	Fringe leaf frog	Present	Metamorphosed	2
Anura	Hylidae	*Hyla*	*chrysoscelis* complex	Gray treefrog complex	Present	Larvae & metamorphosed	2
Anura	Hylidae	*Pseudacris*	*maculata*	Boreal chorus frog	Present	Metamorphosed	2
Anura	Hylidae	*Trachycephalus*	*resinifictrix*	Amazon milk frog	Present	Metamorphosed	1
Anura	Ranidae	*Lithobates*	*pipiens*	Northern leopard frog	Present	Larvae & metamorphosed	1
Anura	Ranidae	*Lithobates*	*sylvaticus*	Wood frog	Present	Metamorphosed	2
Caudata	Ambystomatidae	*Ambystoma*	*laterale*	Blue-spotted salamander	Present	Larvae & metamorphosed	2
Caudata	Ambystomatidae	*Ambystoma*	*maculatum*	Spotted salamander	Present	Metamorphosed	1
Caudata	Ambystomatidae	*Ambystoma*	*opacum*	Marbled salamander	Present	Metamorphosed	1
Caudata	Ambystomatidae	*Ambystoma*	*tigrinum*	Eastern tiger salamander	Present	Larvae & metamorphosed	2
Caudata	Amphiumidae	*Amphiuma*	*tridactylum*	Three-toed amphiuma	Present	Metamorphosed	1
Caudata	Cryptobranchidae	*Cryptobranchus*	*alleganiensis*	Hellbender	Present	Metamorphosed	1
Caudata	Dicamptodontidae	*Dicamptodon*	*ensatus*	California giant salamander	Present	Metamorphosed	1
Caudata	Plethodontidae	*Desmognathus*	*conanti*	Spotted dusky salamander	Present	Metamorphosed	2
Caudata	Plethodontidae	*Eurycea*	*cirrigera*	Southern two-lined salamander	Present	Larvae	2
Caudata	Plethodontidae	*Eurycea*	*guttolineata*	Three lined salamander	Present	Metamorphosed	2
Caudata	Plethodontidae	*Eurycea*	*longicauda*	Long tailed salamander	Present	Metamorphosed	2
Caudata	Plethodontidae	*Hemidactylium*	*scutatum*	Four toed salamander	Present	Metamorphosed	1
Caudata	Plethodontidae	*Plethon*	*glutinosus*	Slimy salamander	Present	Metamorphosed	1
Caudata	Proteidae	*Necturus*	*beyeri*	Gulf Coast waterdog	Present	Larvae	2
Caudata	Salamandridae	*Cynops*	*orientalis*	Chinese fire-belly newt	Present	Metamorphosed	2
Caudata	Salamandridae	*Icthyosaura*	*alpestris*	Alpine newt	Present	Metamorphosed	1
Caudata	Salamandridae	*Notophthalmus*	*viridescens*	Central newt	Present	Metamorphosed	5
Caudata	Salamandridae	*Triturus*	*karelinii*	Southern crested newt	Present	Metamorphosed	1
Caudata	Sirenidae	*Pseudobranchus*	*striatus*	Northern dwarf siren	Present	Metamorphosed	2
Caudata	Sirenidae	*Siren*	*intermedia*	Lesser siren	Present	Metamorphosed	1
Gymnophiona	Typhlonectidae	*Typhlonectes*	*natans*	Rio Cauca caecelian	Present	Metamorphosed	1

Supplementary Figure 1. Phylogenetic relationships among Amphibians from Pyron and Weins (2011) with an emphasis on Caudata (salamanders). The evolutionary relationships for Apoda and Anura are trimmed to a single representative. Taxa within Caudata are trimmed to a representative for each genus, with genera that have been confirmed to be biofluorescent in response to blue excitation wavelengths (440 – 460 nm) (Supplementary Table 1) indicated in green. Terminals and branches indicated in white have unknown biofluorescent properties.

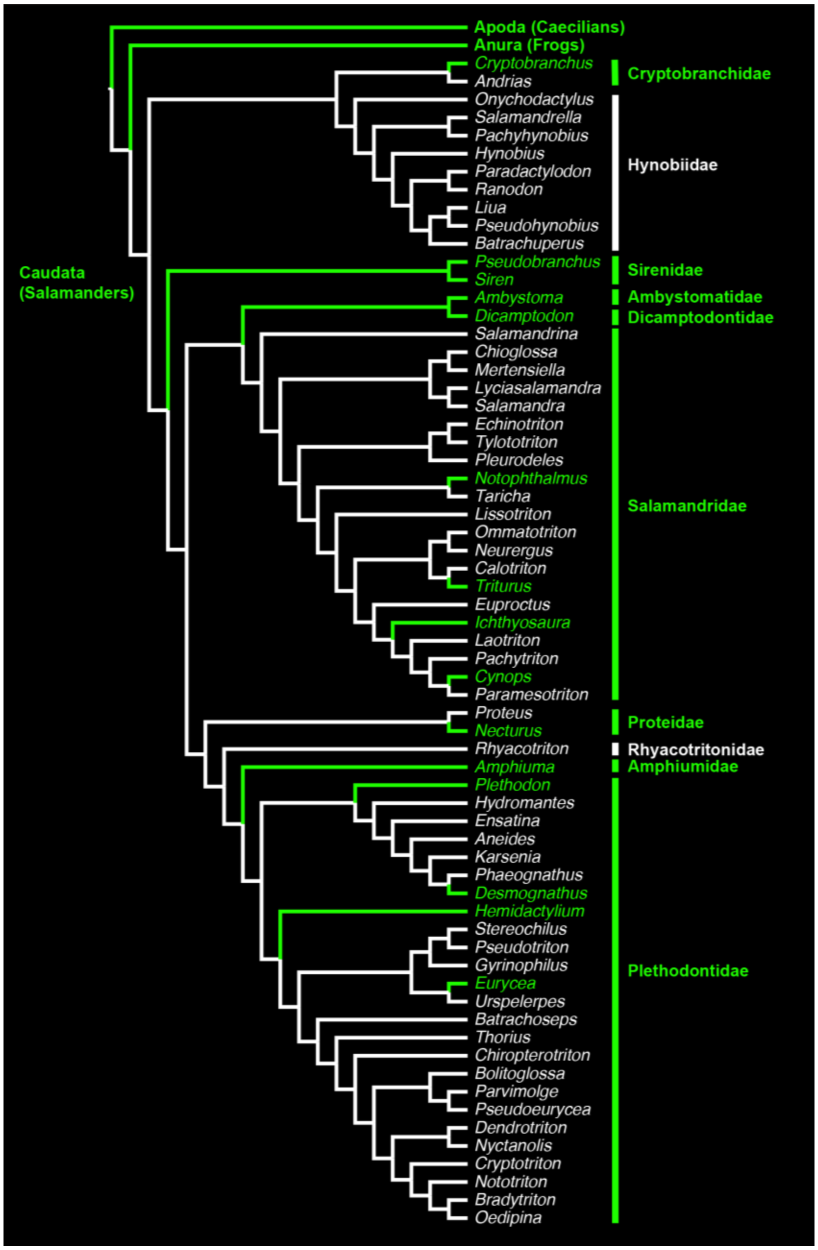

Supplementary Figure 2. Biofluorescent patterns in response to ultra-violet radiation. Imaged are a section of the dorsal surface of an eastern tiger salamander (*Ambystoma tigrinum*) and the posterior dorsal surface of an anuran (*Ceratophrys cranwelli*) fluorescing in response to ultra-violet excitation light. The excitation light used was 360 – 380 nm and we visualized fluorescence via a long pass filter (415 nm). Fluorescence in response to ultra violet light was not as intense as was fluorescence in response to blue light (440 – 460 nm).

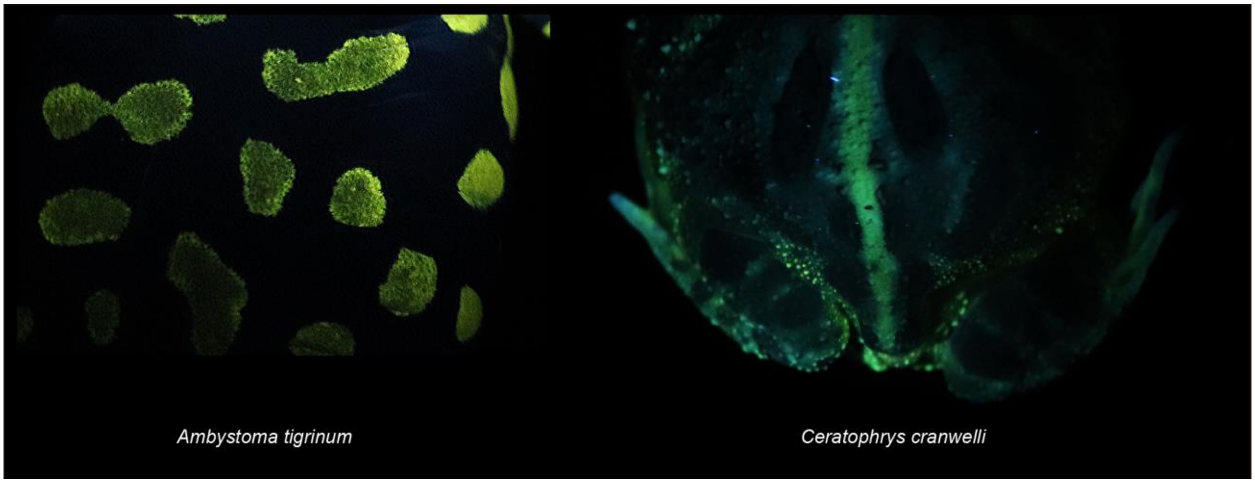

Supplementary Figure 3. Biofluorescent mucous-like secretions and urine. We observed that the secretions or other products of some amphibians biofluoresced in response to blue excitation light (440 – 460 nm). Pictured on the left is the mucous-like secretion from a caecilian (*Typhlonectes*) under white light (top) and then imaged via a blue excitation light and yellow long pass filter (500 nm) (bottom). On the right is a giant salamander (*Dictamptodon*) and its urine imaged with blue excitation light and a yellow long pass filter.

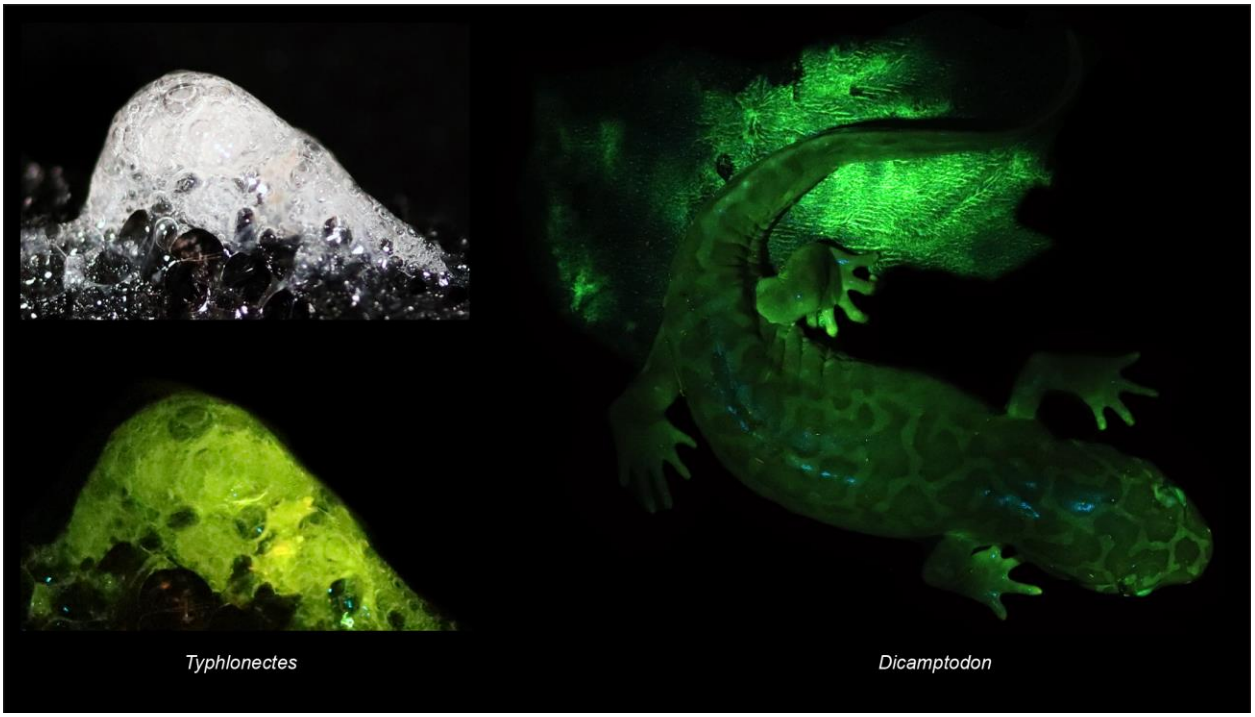

Supplement: Supplementary file 1 — Supplementary materials. [file 41598_2020_59528_MOESM1_ESM.pdf]
